# Alcoholism: effects on the cochleo-vestibular apparatus

**DOI:** 10.1016/S1808-8694(15)31132-0

**Published:** 2015-10-20

**Authors:** Marcieli Bellé, Sílvia do Amaral Sartori, Angela Garcia Rossi

**Affiliations:** 1 M.S Student of Human Communication Disorders - hearing. UFSM; 2 M.S Student of Human Communication Disorders - hearing. UFSM; 3 PhD. Adjunct Professor - Department of Otorhinolaryngology - UFSM

**Keywords:** alcohol

## Abstract

**Summary:**

Several ototoxic drugs are harmful to the human being and lead to problems such as tinnitus, many types of hearing loss, and vertigo. Alcohol is among the main agents considered ototoxic.

**Aim:**

To study the effects of alcoholism in the vestibular-cochlear system.

**Study Design:**

cross-sectional contemporary cohort.

**Materials and Methods:**

The sample comprehended 37 individuals in the Experimental Group, members of Alcoholics Anonymous of the City Santa Maria-RS, and 37 non-alcoholic individuals in the Control Group, age and gender matching. All of the individuals examined were submitted to anamnesis, otorhinolaryngological examination, basic hearing evaluation, and vecto-electronystamography.

**Results:**

67.57% of the individuals from the Experimental Group showed abnormalities in the audiometry and 24.32% presented abnormalities in the computerized vecto-electronystamography. In the Control Group, 27.03% of the individuals showed abnormalities in the audiometry and 10.81% presented abnormalities in the computerized vecto-electronystamography.

**Conclusion:**

Alcohol interferes on an individual's hearing and balance, causing deleterious effects on the human organism.

## INTRODUCTION

The vestibular apparatus is an organ with two functions, the cochlea is responsible for hearing and the labyrinth handles balance. Alterations in some of these senses may bring about great difficulties for a human being, such as reduction in the person's capacity to react to sounds in the environment, reduction in his/her capacity to communicate effectively with the environment, change body balance and generate other problems for the individual.

Dizziness is the major symptom of a balance disorder, that may or may not originate in the vestibular system, and this is only possible to clear up by means of a neurotologic exam. Hearing disorders (dysacusis) and tinnitus may be associated to vertiginous problems^1^.

Many ototoxic drugs cause harmful effects to the human being, such as tinnitus, different hearing deficits and vertigo. Among the many so called ototoxic agents, alcohol is present^2^.

Signs and symptoms frequently found in alcoholism are anorexia, instability and dizziness, nausea, vomit, weight loss, fever and abdominal pain, amongst others.

Some authors believe alcoholism causes premature aging of neuropsychologic functions and possibly affects the brain as well^3^.

It is known that drug use, even therapeutically and the exposure to toxic and chemical substances may cause total or partial loss of the vestibular and/or cochlear functions. Among these exogenous substances is alcohol.

Having seen the deleterious effects of balance and hearing disorders on an individual's life quality, and the impact on his/her social and professional performance, our goal with the present investigation was to study the influences of alcoholism on the vestibulo-cochlear apparatus.

## MATERIALS AND METHODS

This is a contemporary cohort cross-sectional study and is registered at the Projects Department at the Health Sciences Center - UFSM under protocol # GAP/CCS 8063.

Following current ethical concepts in research with human beings, only those individuals who freely agreed to participate in this research were accepted; and after they were informed on the project, they freely signed an informed consent form.

This study was carried out at the Otology Ward of the Santa Maria University Hospital (HUSM/ UFSM) in a group of Anonymous Alcoholics of the City of Santa Maria, RS.

The individuals underwent otorhinolaryngological exam, anamnesis, basic audiologic evaluation and vectoelectronystagmography.

Otorhinolaryngological exam was performed by an ENT physician, aiming at ruling out any ear, nose or throat disorder.

Anamnesis included issues pertaining to alcoholism such as: when he/she started drinking, type of liquor used, amount consumed, abstinence time, family history, as well as hearing and balance complaints; nausea, vomit, unbalance, sleep disorder, headaches, tinnitus and dizziness

Basic audiologic tests battery was made up of Tonal Threshold Audiometry (TTA), Speech Recognition Threshold (SRT), Speech Recognition Percentage Index (SRPI) and acoustic immitance measures (AIM) - encompassing compliance (C), tympanometry (T) and Acoustic Reflex Study, both contra and ipsilaterally^4^.

Vestibular evaluation was carried out by a Contronic SCV 5.0 Computerized Vecto-electronystagmography system. This system carries out vestibulo-oculomotor evaluation by means of a triangular electrode disposition placed near the eyes, which are able to Record cornealretina potential variations during eye movement. Being basically used to record nystagmus, which is the movement of greatest interest in neurotology.

Of the individuals evaluated, we excluded those who abused of other drugs and also those who had any ear, nose and/or throat disorder.

Thus, according to the criteria adopted for sample selection, the experimental group was made up of 37 individuals who belonged to a group of anonymous alcoholics, broken down in two groups, according to age range:

Experimental Group A (EA): made up of 20 (54.05%) individuals, with ages ranging between 33 and 49 years.

Experimental Group B (EB): made up of 17 (45.95%) individuals, with ages ranging between 50 and 70 years.

Following that we collected individuals who did not abuse alcohol, and had gender and age matching each individual from the study, in order to form the control group (37 individuals) and compared them with the experimental group individuals, also broken down according to age range:

Control Group A (CA): made up of 20 (54.05%) individuals, with ages varying between 33 and 49 years.

Control Group B (CB): made up of 17 (45.95%) individuals, with ages varying between 50 and 70 years.

For analysis purposes, data was collected and distributed for each one of the variables studied in Group A (control and experimental) and in Group B (control and experimental). The statistical study was carried out in a descriptive fashion, with the results attained being organized in tables and presented in absolute and relative numbers.

## RESULTS

Table 1 depicts the results attained as to the time of alcohol intake, in years, for individuals from groups EA and EB.

**Table 1:** Distribution of Groups EA and EB individuals in relation to alcohol intake duration, in years.

Alcohol intake duration			
Group	Minimum	Maximum	Mean
EA	5	30	19.5
EB	2	50	29.95

Table 2 depicts the results attained for groups EA and AB individuals, in function of the main complaints reported in the anamnesis.

**Table 2:** Distribution of Groups EA and EB individuals in relation to the main complaints presented during anamnesis.

Group	EA		EB	
	N	%	N	%
Insomnia	6	30.00	9	52.94
Stress	13	65.00	14	82.35
Headache	8	40.00	10	58.82
Irritation	12	60.00	13	76.47
Sleepiness	6	30.00	4	23.53
Depression	12	60.00	13	76.47
Hypertension	2	10.00	3	17.65
Aggressiveness	6	30.00	7	41.17
Forgetfulness	13	65.00	12	70.59
Lack of energy	4	20.00	11	64.70
Lack of attention	7	35.00	8	47.05
Hearing complaint	7	35.00	13	76.47
Hypersensibility to sound	6	30.00	11	64.70

Table 3 depicts the results attained for those individuals in Groups CA and CB, based on their main complaints reported in the anamnesis.

**Table 3:** Distribution of Groups CA and CB individuals in relation to the main complaints presented during anamnesis.

GROUP	CA		CB	
	N	%	N	%
Insomnia	2	10.00	3	17.65
Stress	4	20.00	4	23.53
Headache	2	10.00	3	17.65
Irritation	5	25.00	4	23.53
Sleepiness	1	5.00	1	5.88
Depression	4	20.00	5	29.41
Hypertension	0	0.00	0	0.00
Aggressiveness	0	0.00	1	5.88
Forgetfulness	5	25.00	6	35.29
Lack of energy	0	0.00	5	29.41
Lack of attention	1	5.00	3	17.65
Hearing complaint	2	10.00	7	41.18
Hypersensibility to sound	0	0.00	2	11.76

Table 4 depicts the distribution of individuals from Groups EA and Eb, based on their complaint of tinnitus. The presence of such complaint was signaled through a “Yes” and its absence as a “No”.

**Table 4:** Distribution of Groups EA and EB individuals in relation to tinnitus.

Tinnitus				
	No		Yes	
Group	N	%	N	%
EA	10	50.00	10	50.00
EB	7	41.18	10	58.82
Total	17	45.95	20	54.05

Table 5 depicts the results attained for individuals from Groups CA and CB, based on their tinnitus complaint.

**Table 5:** Distribution of Groups CA and CB individuals in relation to tinnitus.

Tinnitus				
	No		Yes	
Group	N	%	N	%
CA	16	80.00	4	20.00
CB	10	58.82	7	41.18
Total	26	70.27	11	29.73

Table 6 depicts the distribution of individuals from Groups EA and EB based on their dizziness complaint. If present, they marked “Yes”, if absent, they marked “No”.

**Table 6:** Distribution of Groups EA and EB individuals in relation to dizziness.

Dizziness				
	No		Yes	
Group	N	%	N	%
EA	14	70.00	6	30.00
EB	9	52.94	8	52.94
Total	23	62.17	14	37.83

Table 7 presents the distribution of individuals from Groups CA and CB, based on dizziness.

**Table 7:** Distribution of Groups CA and CB individuals in relation to dizziness.

Dizziness				
	No		Yes	
Group	N	%	N	%
CA	18	90.00	2	10.00
CB	13	76.47	4	23.53
Total	31	83.78	6	16.22

Table 8 depicts audiometry results found for individuals from groups EA and EB.

**Table 8:** Distribution of Groups EA and EB individuals in relation to audiometric alterations.

Audiometry				
	Normal		Altered	
Group	N	%	N	%
EA	9	45.00	11	55.00
EB	3	17.65	14	82.35
Total	12	32.43	25	67.57

Table 9 depicts the distribution of individuals from groups CA and CB, based on their audiometric results.

**Table 9:** Distribution of Groups CA and CB individuals in relation to audiometric alterations.

Audiometry				
	Normal		Altered	
Group	N	%	N	%
CA	16	80.00	4	20.00
CB	11	64.71	6	35.29
Total	27	72.97	10	27.03

Table 10 depicts the results attained for groups EA and EB individuals in computerized vecto-electronystagmography

**Table 10:** Distribution of Groups EA and EB individuals in relation to VENG alterations.

VENG				
	Normal		Altered	
Group	N	%	N	%
EA	17	85.00	3	15.00
EB	11	64.70	6	35.30
Total	28	75.68	9	24.32

Table 11 depicts the distribution of individuals from groups CA and CB, based on them having or not alterations in computerized vecto-electronystagmography.

**Table 11:** Distribution of Groups CA and CB individuals in relation to VENG alterations.

VENG				
	Normal		Altered	
Group	N	%	N	%
CA	19	95.00	1	5.00
CB	14	82.35	3	17.65
Total	33	89.19	4	10.81

Table 12 depicts the results attained in audiometry and computerized vecto-electronystagmography for individuals from groups EA and EB.

**Table 12:** Distribution of Groups EA and EB individuals in relation to audiologic and vestibular findings.

GROUP	Normal hearing X Normal VENG		Normal hearing X Altered VENG		Altered hearing X Normal VENG		Altered hearing X Altered VENG	
	N	%	N	%	N	%	N	%
EA	9	45,00	0	00,00	8	40,00	3	15,00
EB	1	5,88	2	11,76	10	58,82	4	23,52
Total	10	27,03	2	5,40	18	48,65	7	18,92

## DISCUSSION

On Table 1, one can see that the individuals from group EA presented average duration of alcohol intake equal to 19.5 years, and individuals from group EB had 29.45 years. When we compared the two groups, we notice that the individuals from group EB had average alcohol intake duration of 10.45, longer than that of individuals from group EA.

Results related to the complaints presented at the anamnesis by Groups EA and EB individuals are depicted on Table 2. As to the main complaints reported by the individuals from each group, from Group EA we highlight: stress (65%), forgetfulness (65%), irritation (60%) and depression (60%). For group EB, the main complaints were: stress (82.35%), irritation (76.47%), hearing complaint (76.47%), depression (76.47%), forgetfulness (70.59%), lack of energy (64.7%), hypersensibility to sound (64.7%), headache (58.82%) and insomnia (52.94%).

Table 3 depicts the results attained for Groups CA and CB individuals, based on their complaints collected during the anamnesis. For group CA individuals we may highlight: stress (20%), forgetfulness (25%), irritation (25%) and depression (20%). For individuals from the CB group, the main complaints were: stress (23.53%), irritation (23.53%), hearing complaint (41.18%), depression (29.41%), forgetfulness (35.29%) and lack of energy (29.41%).

When we compare the results attained in tables 2 and 3, we see that the complaints collected during anamnesis were present in most individuals from the experimental group, either EA or EB.

Many authors from the literature relate psychological symptoms to alcohol abuse. When he studied patients with psychiatric disorders and that had drank alcoholic beverages for 15 to 20 years, Lima^13^ concluded that chronic alcoholism compromises an individual's mental, physical and social performance. Other authors describe in their studies the occurrence of psychological symptoms because of the abusive consumption of alcoholic beverages^14^, ^15^, ^16^, ^17^, ^18^.

The results attained in the present investigation are in agreement with those from the aforementioned authors - Table 1 shows that individuals from Group EB had average duration of alcohol consumption above those from individuals belonging to Group EA, in 10.45 years; and thus, shown in Table 2, there was a higher prevalence of all analyzed complaints in individuals from group EB, corroborating the statement that the abuse of alcohol causes not only physical symptoms, such as alterations in different organs and systems, but also psychological symptoms.

Table 4 depicts the tinnitus complaint in individuals from the Experimental Group. In individuals from Group EA, tinnitus was mentioned by 50% (N=10) of the individuals; now, on group EB, 58.82% (N=10) of the individuals complained of it.

On Table 5 it is possible to see the results attained for individuals from Control Groups A and B as far as tinnitus is concerned. Such complaint was present in 20% (N=4) of the individuals from Group CA and in 41.18% (N=7) of those from Group CB.

Comparing Tables 4 and 5, we notice that tinnitus was present in both the Experimental and the Control Groups, with a higher prevalence in those individuals from the Experimental Group, both EA and EB.

When we compare this analysis to what has been published in the literature, we can see an agreement with papers from authors who stated that tinnitus may have numerous causes, such as ototoxic drugs - like alcohol, that may trigger such disorder^9^,^12^,^19^, ^20^, ^21^.

Table 6 depicts the presence of diziness in the Experimental Group. In individuals from Group EA, diziness was mentioned by 30% (N=6) of the them; and in Group

EB, this complaint was reported by 52.94% (N=8) of the individuals evaluated. When we analyze Table 6, it is possible to see that dizziness was present in both groups; notwithstanding, in Group EB, this complaint was present in more than half of the individuals.

Table 7 depicts the results found for the Control Group as to diziness. For individuals from Group CA, such complaint was present in 10% (N=2) of the patients and for Group CB it was present in 23.53% (N=4).

Comparing Tables 6 and 7, it is possible to stress that dizziness was present in both the experimental and the control groups; however, it was also more evident in the experimental group, both for EA and EB.

Some studies state that many drugs, alcohol included, cause dizziness as side effect, and thus negatively influence motor skills, including tasks with simple time of reaction, coordination skills, balance and hand-eye coordination^10^,^15^,^18^,^21^, ^22^, ^23^, ^24^, ^25^. The findings from this study are in agreement with those from the literature studied.

Audiometric results as to the presence or not of hearing alterations, in the experimental group, are depicted on Table 8. We have seen that 55% (N=11) of the individuals from Group EA had hearing impairment, and 82.35% (N=14) of the individuals from Group EB presented this alteration. We may see that in Group EB, most individuals had some hearing loss.

Table 9 depicts the audiometric results found for individuals in the Control Group. For the CA Group, 20% (N=4) of the individuals presented some alteration in this exam. In individuals from group CB, 35.29% (N=6) presented such alteration.

Papers from some authors state that toxic substances such as alcohol cause hearing alterations as side effect, causing degenerative lesion in the hair cells of the Corti Organ^5^,^7^,^20^,^21^,^25^,^26^. Comparing data from Tables 8 and 9 and data from the literature, we notice that alcoholic individuals have hearing loss, and those that consumed alcohol for longer periods had higher incidences of hearing loss. The findings of the present study corroborate those from the authors we consulted.

When we compare the results attained on Tables 4 and 5, we can see that hearing loss was higher than tinnitus as a complaint, for both EA and EB groups. Authors such as Silva, Munhoz, Ganança & Caovilla20 reported that most drugs are deleterious for the cochlea, first compromising the base cells, affecting the higher frequencies. Thus, because of such alteration, there is a close relationship between hearing loss and tinnitus, and the latter is an adjuvant symptom in high frequency hearing loss.

Results from the computerized vectoelectronystagmography for groups EA and EB individuals may be seen on Table 10. We noticed that 15% of Group EA individuals (N=3) and 35.3% (N=6) of Group EB individuals had alterations in vectoelectronystagmography. We then stress that such alteration was more evident in Group EB individuals.

Table 11 depicts the results found in the computerized vectoelectronystagmography for Groups CA and CB individuals. It was possible to notice that 5% (N=1) of individuals from Group CA and 17.65% (N=3) from group CB had alterations in the computerized vectoelectronystagmography.

Comparing results from Tables 10 and 11, it is possible to see that the alterations were more evident in the individuals from the Experimental Group, both EA and EB, and the latter had the higher number.

Many authors from the specialized literature reported results similar to the ones found in the present study, mentioning that ototoxic agents, such as alcohol, have a negative impact on the vestibular apparatus, causing vertigo and dizziness^6^,^7^,^8^,^20^,^27^.

Table 12 depicts the relationship among results attained in the audiologic evaluation and the results from vectoelectronystagmography (VENG) for both experimental groups: Groups EA and EB. As to Group EA, the individuals obtained the following results: 45% (N=9) of the individuals presented normal hearing and normal VENG result; and 40% (N=8) had altered hearing and normal VENG. As to individuals from the EB group, we have observed that 58.82% (N=10) had altered hearing and normal VENG; and 23.52 (N=4) had alterations in both hearing and VENG.

When we consulted the specialized literature, some authors mention that alcohol is considered an ototoxic agent, have a deleterious effect on the whole body, and it may even cause hearing and vestibular alterations^5^,^7^,^8^,^9^,^12^.

Authors have reported that alcohol may cause alterations to both the hearing and/or the vestibular systems^6^,^10^,^11^,^20^,^25^,^27^. According to Silva, Munhoz, Ganança & Caovilla20 most drugs are deleterious to the cochlea, and less so to the labyrinth. This study agrees with the aforementioned authors, having seen that the results have proven that alcohol first influences the auditory system and later the vestibular system.

## CONCLUSION

In this investigation it was possible to observe that individuals who abused alcohol had lower results than the individuals in the control group, in all tests applied. Thus, in the present study we can conclude that alcohol intake interferes in hearing and in an individual's balance, bringing about a deleterious effect to the human body.
